# Influence of Various Acidic Beverages on Tooth Erosion. Evaluation by a New Method

**DOI:** 10.1371/journal.pone.0129462

**Published:** 2015-06-02

**Authors:** Stefan Zimmer, Georg Kirchner, Mozhgan Bizhang, Mathias Benedix

**Affiliations:** Dept. of Operative and Preventive Dentistry, School of Dentistry, Witten/Herdecke University, Alfred-Herrhausen-Str. 50, Witten, Germany; Glasgow University, UNITED KINGDOM

## Abstract

**Material & Methods:**

We have analyzed the loss of enamel and dentine after exposure to different non-alcoholic drinks with a simple new method using bovine teeth. 100 enamel and 100 dentine specimens from freshly extracted bovine incisors were randomly attributed to 10 groups (n=10 for enamel and dentine each). Prior to the start of the experiment all specimens were weighed using a precision balance. The mean initial masses (SD) were 35.8 mg (7.2) for enamel and 24.7 mg (7.0) for dentine. No statistically significant differences were found between groups for initial masses (p>0.05, ANOVA with Bonferroni post hoc test). Thereafter, all specimens of one group were simultaneously placed in 200 ml of the following fluids: Coca-Cola, Coca-Cola light, Sprite, apple juice, Red Bull, orange juice, Bonaqua Fruits (Mango-Acai), tap water, chlorinated swimming pool water, and lemon juice. Fluids were continuously ventilated at 37° C for 7 days. Thereafter the specimens were weighed again and the mean mass loss was calculated.

**Results:**

The values were (enamel/dentine): Coca-Cola 7.5 mg/6.6 mg; Coca-Cola light 5.2 mg/3.5 mg, Sprite 26.1 mg/17.7 mg, apple juice 27.1 mg/15.2 mg, Red Bull 16.6 mg/17.0 mg, orange juice 24.3 mg/20.2 mg, Bonaqua Fruits (Mango-Acai) 17.8 mg/16.2 mg, tap water -0.2 mg/-0.3 mg, swimming pool water -0.3 mg/-0.2 mg, and lemon juice 32.0 mg/28.3 mg. From all drinks, Cola and Cola light showed the least erosivity (p<0.001, ANOVA with Bonferroni post hoc test) whereas lemon juice showed statistically significant higher erosivity than all other drinks except Sprite and apple juice (p<0.01, ANOVA with Bonferroni post hoc test).

**Conclusions:**

In conclusion, erosivity of common non-alcoholic drinks varies widely. For example, Sprite, apple juice, and orange juice are about five times more erosive than Coca-Cola light. The findings from the present study should be taken into account in choosing a diet that provides satisfactory nutrition while minimizing tooth erosion.

## Introduction

Tooth erosion is a chronic loss of dental hard tissues (enamel and dentine) caused by acids of intrinsic (gastric) or extrinsic (dietary) origin. To some extent, it is a physiological and age dependent process related to acid containing food intake [1]. A pathological status is reached at the latest when the teeth are so worn that their functionality is impaired [2], but it may also be already perceived by patients and dentists when the appearance of the teeth is affected [1]. In contrast to the physiological status of erosion, no age dependence is found in the pathological form [1]. It is classified as a disease in the WHO ICD10-classification [3].

The prevalence of tooth erosions is high and continuously growing within populations [4]. A recent study from Israel [5] showed a prevalence of between 36.6% in 15-18-year-olds and 61.9% in 55-60-year-olds. In their systematic review, Salas et al. showed a prevalence of 30.4% in 8-19-year-old children and adolescents [6]. Although the prevalence of tooth erosion is high and increasing in many countries, its relevance for oral health is not comparable to caries and periodontitis. For example, no tooth loss is reported due to tooth ersosion in Germany, but 14.1 teeth are lost on average due to caries and periodontitis at the age of 65–74 years [7].

Since no age dependence is found [1], an independent variable may be responsible for severe levels of erosive destruction. Mulic et al. found that gastric acids (reflux or vomiting), fruit juices and soft drinks are risk indicators for erosive wear [8]. This was confirmed in a meta-analysis by Hi et al. where soft drinks showed the highest and statistically significant odds ratio (2.41) for the development of dental erosions [9]. In accordance, Habib et al. found a statistically significant odds ratio of 2.38 for acidic fruit juice consumption [10]. Since the consumption of soft drinks is continuously increasing in developed countries [11], the knowledge about their erosivity is important in dietary and dental counseling. However, Barbour and Lussi stated that “a ranking for the in vivo erosivity of different acidic drinks…is rather complicated if not impossible” [12]. The need for valid data on the erosivity of soft drinks having been recognized, the question arises which methodology could be used to address this problem. To date, several qualitative and quantitative *in-vivo*, *in-situ* and *in-vitro* methods have been used to determine tooth erosivity in enamel and dentine [13,14]. For diet counseling, only quantitative data are relevant. According to Schlueter et al., profilometry is the most commonly applied quantitative method, followed by measurement of surface hardness and microradiography. Measurement of calcium and phosphate release from dental hard tissues in solutions is another common method [13].

Non-contact, 3-D-profilometry is a valid tool in determining surface tissue loss, but it does not allow to measure the mineral loss occurring beneath a pseudo-intact surface [13]. This is possible by microradiography but, like 3-D-profilometry, specimen preparation and analysis are time consuming and require expensive technical equipment. Measuring surface hardness is at least a simple and quick method, but can only show the status of the remaining surface without giving information about lost hard tissue at the surface of the specimen.

A possible simple and meaningful method may be gravimetric analysis. This method evaluates the erosivity by weighing enamel or dentine specimens before and after erosive exposition. To our knowledge, the first and only studies using a gravimetric method were performed by von Fraunhofer and Rogers using a small number of human tooth samples [15,16]. In consideration of the described needs and methodologies, it was the aim of the present study to generate some data for commonly consumed non-alcoholic beverages with a newly developed gravimetric method using bovine tooth specimens. Additionally, the erosivity of chlorinated swimming pool water should be analyzed since pathologic erosion was observed in competitive swimmers [17]. Both aims could be achieved.

## Materials and Methods

This was an *in-vitro* study on bovine enamel and dentine specimens. All animal material (teeth) used in this study originated from the slaughterhouse Schlachthof Bochum GmbH, Freudenbergstr. 45K, 44809 Bochum—Hamme / Germany. All cattle were slaughtered for meat production and not for research purposes.

**Fig 1:**
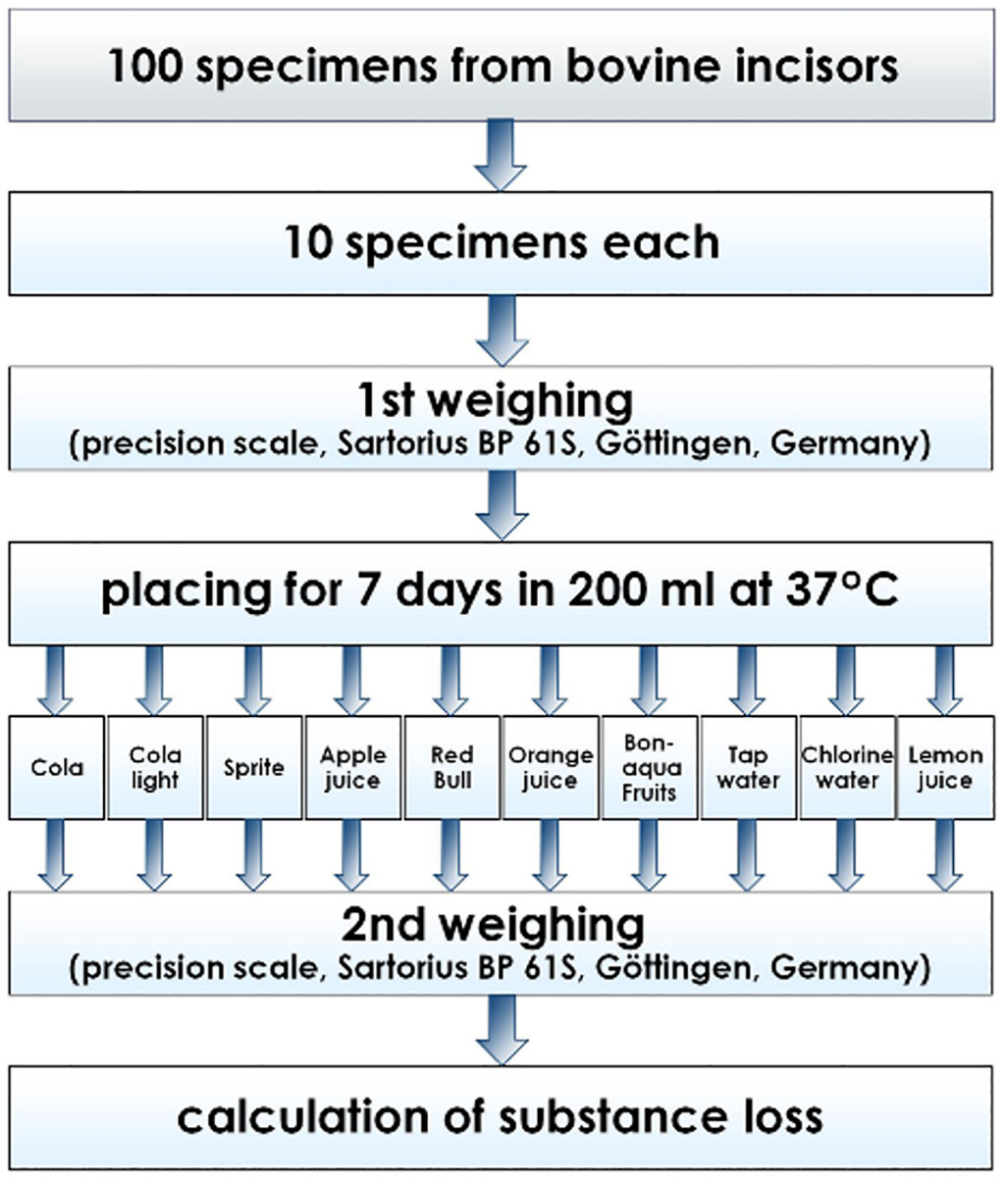
Flow diagram of the study design. The study protocol was conducted twice, separately for enamel and dentine.

Before the main experiment was started, a preliminary test was performed with ten enamel and dentine specimens each to validate the study design and to determine an adequate exposure time. In the main experiment, similar enamel and dentine specimens (n = 200, 5 mm Ø) were gathered from freshly extracted bovine incisors and ground down to a thickness of 1 mm. Specimens were randomly attributed to 10 groups (n = 10 enamel and dentine specimens each). Prior to the start of the experiment all specimens were dried on blotting-paper at room temperature for one hour and weighed using a precision balance (Sartorius BP61S, Göttingen, Germany, metering accuracy 0.1 mg). The mean initial masses (SD) were 35.8 mg (7.2) for enamel and 24.7 mg (7.0) for dentine. No statistically significant differences were found between groups for initial masses (p>0.05, ANOVA with Bonferroni post hoc test). Thereafter, all specimens of one group were simultaneously placed in a pvc pannier which was suspended in a plastic container containing 200 ml of the following fluids: Coca-Cola, Coca-Cola light, Sprite (all Coca-Cola Erfrischungsgetränke AG, Germany), apple juice (Amecke Fruchtsaft GmbH, not-from-concentrate juice, clear), Red Bull (Red Bull GmbH, Germany), orange juice Direktsaft (real, -Handels GmbH), Bonaqua Fruits (Mango-Acai) (Coca-Cola Erfrischungsgetränke AG, Germany), tap water (Chlorine free, disinfected with UV-irradiation, public water supply, Witten/Germany, fluoride concentration <0.25 ppm), swimming pool water from a local public swimming pool (Witten-Annen/Germany), and lemon juice (Sportfit Fruchtsaft GmbH & Co KG). According to the product declarations, none of the commercial products contained any preservatives. Tap water served as a negative, lemon juice as a positive reference. Fluids with specimens were continuously ventilated by an aquarium pump at 37°C for 7 days. The air outlet was placed at the bottom of the containers to ensure a good aeration from the ground to the surface of the fluids. The fluids were replaced daily. Thereafter the specimens were removed from the fluids, rinsed with saline solution for 30 seconds and dried on blotting-paper at room temperature for one hour. Subsequently they were weighed again and the mass loss was calculated in mg. The pH values of all liquids were determined (Beckmann Coulter GmbH Krefeld, Germany, Serial# 0217107). Fig 1 shows the flow diagram of the study design which was carried out twice, separately for enamel and dentine.

Since a Kolmogorov Smirnov test showed normal distribution, means and standard deviations were calculated. One way ANOVA with Bonferroni post hoc test was used to compare the results between groups.

## Results

Table 1 shows the mean (SD) mass losses of enamel and dentine after seven days as well as the results of the pH measurement. Coca-Cola and Coca-Cola light showed the least erosivity (p < 0.001) with respect to enamel and dentine. For dentine, lemon juice showed statistically significant higher erosivity than all other liquids (p < 0.001). This was also true for enamel except for Sprite and apple juice (p < 0.01). No statistically significant differences were found between the other liquids

**Table 1:** Mean weight loss of enamel and dentine after seven days and initial pH of liquids.

	Hard tissue loss in mg (mean, SD)		
	Enamel^1^	Dentine^2^	Initial pH
**Coca-Cola**	**7.5 (0.6)a**	**6.6 (0.7)a**	**2.47**
**Coca-Cola light**	**5.2 (0.8)a, d**	**3.5 (0.8)a, c**	**2.59**
**Sprite**	**26.1 (7.1)b, c**	**17.7 (3.4)b**	**2.68**
**Apple juice (Amecke)**	**27.1 (6.1)b, c**	**15.2 (4.6)b**	**3.38**
**Red Bull**	**16.6 (2.9)b**	**17.0 (1.8)b**	**3.38**
**Orange juice (real)**	**24.3 (4.9)b**	**20.2 (4.8)b**	**3.87**
**Bonaqua Fruits(Mango Acai)**	**17.8 (1.7)b**	**16.2 (3.9)b**	**3.63**
**Tap water**	**-0.2 (0.3)d**	**-0.3 (0.3)c**	**7.40**
**Chlorinated swimming pool water**	**-0.3 (0.3)d**	**-0.2 (0.4)c**	**7.31**
**Lemon juice (Sportfit)**	**32.0 (5.7)c**	**28.3 (6.7)**	**2.50**

^1^Enamel: Values with the same letters are not significantly different. Cola and Cola light showed significantly lowest values (p < 0.001) of all drinks,lemon juice showed significantly higher erosivity than all other drinks except Sprite and apple juice (p < 0.01).

^2^ Dentine: Values with the same letters are not significantly different. All other values are significantly different at p < 0.001

## Discussion

In the present study, specimens from bovine teeth were used to analyze the erosivity of several liquids, mainly soft drinks und fruit juices. Compared to human teeth, bovine teeth are easier to gain and they have a known and homogenous history. All teeth originated from the same cattle herd, all cattle received the same feed and were at the same age when they were slaughtered. There was no noteworthy fluoride exposure from food or water. In addition, bovine teeth are much bigger than human teeth and therefore it is easier to prepare suitable specimens. There are no ethical concerns in contrast to the use of extracted human teeth. Nor is it possible to get a homogenous sample of extracted human teeth. They are largely varying in age, fluoride exposure and were subjected to different diets. Furthermore, it has been shown that bovine teeth are an equivalent substitute to human teeth for erosion studies [18]. All specimens used in the present study had approximately the same diameter (5 mm), thickness (1 mm) and therefore the same surface (55.0 mm^2^) allowing the same exposure to the tested liquids.

This study provides no absolute data on the hard tissue loss caused by tooth erosion in the oral cavity because individual host factors such as saliva composition and flow rate as well as individual drinking habits have a strong influence on this outcome [19,20]. Therefore, it was not intended to simulate natural conditions of the oral cavity. The aim of this study was to provide a relative value of erosivity for the tested soft drinks in the sense of a material property. For diet counseling the data will allow a ranking for the most erosive beverages compared to the erosivity of tap water. However, it has to be considered, that the results of the present study are only valid for the tested brands of soft drinks and juices. Other brands may have a different erosivity.

Daily counseling of erosive drinks is often misleadingly based on the pH-value of the respective products. However, the pH alone gives no valid information about the erosivity of drinks. Although enamel may be dissolved at a pH of 5.2–5.9 [21] and dentine already at pH 6.0–6.8 [22] there is no fixed critical pH for dental erosion [19]. Besides pH, factors such as acid type (e.g. phosphoric acid or citric acid), buffer capacity, adhesion, chelating effect, phosphate-, fluoride- and calcium content of the drink play a role for the erosive properties of a drink. [19] Neither is it enough to know the titratable acid of a drink to judge its erosive capacity. In contrast, the gravimetric method used in the present study is suitable to include all these properties. However, it is not suitable to include patient related factors such as drinking habits, saliva composition and flow rate, and oral hygiene measures (e.g. fluoride administration). These factors can only be determined in the framework of a meticulous dental examination and risk assessment and are not a part of diet counseling.

In their gravimetric study on dental enamel, von Fraunhofer and Rogers used two human specimens per beverage in a 14-day study. Their results were in good accordance with the present study. From all soft drinks, Coca-Cola showed the lowest (2.78 mg / cm^2^), Sprite an average (8.60 mg / cm^2^) and Diet Mountain Dew (14.82 mg / cm^2^) the highest substance loss. The same as in our study, tap water showed no erosivity (-0.05 mg / cm^2^) [15]. Jensdottir et al. used a different method in their study on five cola drinks and five orange juices [23]. At the beginning of the experiment, the drinks were titrated with 1 M NaOH until a pH of 5.5 was reached. The titratable acid for the orange juices was nearly six times higher than for the cola drinks. Thereafter, 50 mg hydroxyapatite (HAP) powder was added to 50 mL of the original solutions. The dissolved HAP was determined and an “erosive potential” was calculated as mg HAP dissolved per liter of drink after three and 30 minutes. Within the first minute of exposure, cola drinks showed a higher erosive potential than orange juices, probably due to their much lower pH (2.70 vs. 3.73). After three minutes, however, the erosive potential in the cola drinks slowed to less than one fortieth whereas it slowed to less than one third in the orange juices. This shows that properties other than pH, such as buffering power, kind of acid, and chelating effect of the drinks have a strong impact on their erosivity and that the mainly phosphoric acid containing cola drinks are saturated faster with respect to HAP than the citric acid contained in orange juices. In the present study the erosivity was only determined after seven days and not after three minutes. This was done on the basis of the preliminary study in order to allow a good discrimination between the tested liquids. It is clear that such a long exposure time does not correlate with a single drink consumption. However, if the exposure time of the teeth to acidic drinks is five minutes per day only, the seven days from the present study reflect a life time of 5.5 years.

Jager et al. determined the erosive potential of soft drinks as loss of calcium to the beverage after different exposure times. From the calcium concentrations found in the beverages by atomic absorption spectrometry, they calculated the enamel loss of the specimens in μm. After three minutes Coca-Cola showed a loss of 0.34 μm, apple juice 1.06 μm, and Sprite the highest value (3.74 μm) [20]. With respect to the ranking of the three drinks, the results remained stable during the entire study period of 30 minutes. However, as already shown in the short-term study of Jensdottir et al. [23], the erosivity of the drinks on enamel developed differently over time. After 30 minutes of exposure, Coca-Cola showed a loss of 1.18 μm, apple juice 3.81 μm, and Sprite 5.34 μm. In contrast to the present study, the erosivity of Sprite was still higher than those of apple juice at the end of the study. The reason may be the different variety of apple juices, but also the different exposure times of both studies. Having in mind the results of Jensdottir et al. and the development of erosive potential between three and 30 minutes exposure time in the study of Jager et al., it might be speculated that the erosive potential of Sprite and apple juice might be equivalent after seven days as found in the present study.

Although pathologic erosion was observed in competitive swimmers [17], the erosive potential of swimming pool water did not differ from tap water. However, it must be considered that the teeth of competitive swimmers are exposed to water for hours every day. Since water is also undersaturated with respect to the minerals of dental hard tissues, there is also dissolution of enamel in water as a function of time [24]. Enamel and dentine specimens from tap water and pool water showed a small mass increase during the study (0.2 and 0.3 mg). This might be attributed to some water absorption of the specimens.

Although dentine dissolves already at a higher pH than enamel, the present study showed more substance loss for enamel. This can be explained by the fact that dentine has a much higher content of organic material (24.69 weight %) than enamel (0.98 weight %), primarily in the form of collagen [25]. This collagen network is not dissolved by acid and may have a sealing effect on the exposed dentine surface preventing further dissolution of mineral after a certain time. This is supported by the fact that a soft covering was found at the dentine surface after seven days exposure time. Therefore, a shorter exposure time for dentine might be discussed for future studies. However this does not mean that the smear layer might prevent further dentine loss in the *in-vivo*-situation since it will be brushed away by daily oral hygiene.

## Conclusions

Erosivity of common non-alcoholic drinks as measured in vitro by substance loss of bovine enamel and dentine varies widely. For example, Sprite, apple juice, and orange juice are about five times more erosive than Coca Cola light. The findings from the present study may be helpful in daily dietary and dental counseling since they allow identifying the most erosive non-alcoholic beverages. However, it has to be considered by dental professionals, that not only dental aspects are important in nutrition counseling. Despite its high erosivity, orange juice may be a valuable contribution to a healthy nutrition whereas other non alcoholic drinks with low erosivity are not.
